# Fair access to higher surgical training in the UK: an equity, diversity and inclusion analysis of national selection in 2024

**DOI:** 10.1136/bmjopen-2025-106487

**Published:** 2025-11-13

**Authors:** Jaspreet Kaur Seehra, Ricky Ellis, Brett Doleman, Esther McLarty, Jonathan Lund

**Affiliations:** 1 University of Nottingham Faculty of Medicine and Health Sciences, Nottingham, England, UK; 2 University of Aberdeen, Aberdeen, Scotland, UK; 3 Department of Urology, University Hospitals Plymouth NHS Trust, Plymouth, England, UK

**Keywords:** Education, Medical, MEDICAL EDUCATION & TRAINING, SURGERY

## Abstract

**Abstract:**

**Objectives:**

To assess the impact of gender, age, ethnicity and country of primary medical qualification (CoQ) on outcomes in the 2024 UK ST3 surgical national selection process.

**Design:**

Retrospective cross-sectional analysis of national recruitment data.

**Setting:**

UK-wide ST3 surgical training recruitment.

**Participants:**

2009 unique ST3 applicants to six surgical specialties (otolaryngology, plastic surgery, urology, paediatric surgery, trauma and orthopaedics and general surgery); neurosurgery, cardiothoracic surgery and oral and maxillofacial surgery were excluded.

**Primary outcome measure:**

Offer of a ST3 National Training Number (NTN).

**Results:**

CoQ was the strongest factor associated with success. International medical graduates had lower odds of receiving offers in all specialties, notably in general surgery (adjusted OR (aOR)=0.21, 95% CI 0.14 to 0.33, p<0.001), and trauma and orthopaedics (T&O) (aOR=0.14, 95% CI 0.08 to 0.23, p<0.001). Older age reduced odds in T&O (aOR=0.44, 95% CI 0.26 to 0.72, p=0.001). Female applicants in T&O also had lower odds of success (aOR=0.44, 95% CI 0.25 to 0.79, p=0.006). Ethnicity was not independently associated with outcomes after adjustment.

**Conclusions:**

ST3 selection outcomes are primarily associated with CoQ. UK-trained applicants have a consistent advantage. Women remain less likely to be offered an NTN than men in Trauma and Orthopaedics. This analysis enables detailed and timely equity monitoring across surgical specialties and flags areas for intervention.

**STRENGTHS AND LIMITATIONS OF THIS STUDY**

- Uses a single national dataset covering UK-wide 2024 ST3 recruitment across six surgical specialties.
- Applies consistent outcome definitions with specialty-stratified analyses and a pooled secondary model.
- Multivariable adjustment is based on a directed acyclic graph to prioritise confounding control.
- Specialty-level sample sizes limit power for some subgroups and interactions.
- Visa/immigration status, disability and socioeconomic variables were unavailable, restricting adjustment for potentially important confounders.

## Introduction

 UK national selection for ST3 surgical training was introduced in 2010 to promote fairness and standardisation following MMC reforms.^1^ The process is coordinated centrally by the Medical and Dental Recruitment and Selection (MRDS) service under NHS England Workforce, Training and Education (WTE) on behalf of the four UK nations.^2^ Applications are anonymised and assessed using standardised portfolio scoring and structured, multi-station interviews.^2^ Final offers are made in national rank order through a single, centralised system designed to promote consistency and transparency across specialties.^3^

Equity in postgraduate surgical recruitment is imperative for the NHS, as an inclusive and representative workforce has been shown to enhance patient care, improve team performance and strengthen public relations and confidence in the healthcare system.^4 5^ Conversely, perceived or actual unfairness in recruitment can contribute to differential attainment, lower morale and attrition of minoritised groups.^6 7^ For these reasons, the General Medical Council (GMC) and NHS England have prioritised reducing disparities in training outcomes and eliminating barriers to progression for doctors from diverse backgrounds, particularly in surgical specialties, where representation gaps have historically persisted.^8 9^

Previous research has identified potential disparities in recruitment and career progression across several protected characteristics, including gender, age, ethnicity and country of medical qualification.^10^,^12^ Evidence suggests that certain minoritised groups, such as women, ethnically diverse and internationally trained doctors, may face systemic barriers at various stages of training and assessment, contributing to unequal outcomes.^10 12 13^

In 2024, a new data-sharing agreement between NHS England WTE and JCST enabled access to national-level data with detailed demographic breakdowns. This allowed for timely and accurate analysis of equity in ST3 surgical selection.

This study aims to evaluate whether protected characteristics, including gender, age, ethnicity and country of primary medical qualification, are associated with differential outcomes in the ST3 national selection process for higher surgical training in the UK.

## Methods

### Study design

A retrospective cross-sectional analysis from the 2024 national selection cycle for ST3-level higher surgical training posts was performed. A single year (2024) was specifically chosen to assess the current climate and recent practices within the selection process, providing a timely and relevant snapshot of equity and diversity. The dataset was obtained from the Medical and Dental Recruitment and Selection (MRDS), which operates under the Workforce, Training and Education (WTE) directorate of Health Education England (HEE) and runs national selection processes on behalf of the statutory education bodies of the four home nations of the United Kingdom. The analysis used fully anonymised data supplied under a data-sharing agreement between NHS England WTE and the JCST. In accordance with the guidance of the University of Nottingham Faculty of Medicine and Health Sciences Research Ethics Committee, this secondary analysis of anonymised administrative data did not require Research Ethics Committee review.

### Study population

The analysis was conducted on a pan-specialty basis and included data from six surgical specialties with more detailed subgroup analysis for otolaryngology (Ear, Nose and Throat - ENT), plastic surgery, urology, paediatric surgery, trauma and orthopaedics (T&O) and general surgery. Neurosurgery, OMFS and cardiothoracic surgery were excluded due to different recruitment structures or small numbers. Where key demographic data were missing, the affected applicant was excluded from the relevant analysis.

Variables relating to visa or immigration category were not available and therefore could not be adjusted for, which may limit interpretation regarding overseas applicants’ eligibility for training posts. However, defining the outcome as ‘offer made’ rather than ‘offer accepted’ mitigates potential bias introduced by such post-offer constraints. Applicants could apply to more than one speciality. Where duplicates occurred, only the earliest application per applicant was kept, ensuring independence of observations.

### Variables and outcome measure

The primary outcome was receipt of a training offer across six surgical specialties, defined as ‘offer made’ including offers that were accepted, declined, withdrawn or expired. This broader definition captures the point of selection success and avoids bias introduced by applicant side factors such as programme preferences, availability or visa restrictions. Using ‘offer made’ ensures that applicants who were offered posts but subsequently could not accept them for administrative reasons, such as right-to-work or visa eligibility, are appropriately represented in analyses of selection fairness.^14 15^ A sensitivity analysis using ‘offer accepted’ as the outcome was performed to confirm the robustness of findings (online supplemental table S1).

Main predictor variables included gender, age, ethnicity and country of primary medical qualification. Ethnicity was further categorised into White, Asian, Black, multiple/mixed or other/Chinese trainees, according to the UK Office for National Statistics (ONS) guidelines.^16^ Descriptive statistics were calculated for specialty by region, gender distribution, academic versus clinical placement breakdown and proportion of applicants with a UK versus non-UK primary medical qualification.

### Statistical analysis

Multivariable models were informed by a directed acyclic graph (DAG; online supplemental figure S1) developed to identify key confounders and avoid over-adjustment or collider bias.^17 18^ The DAG was used to represent hypothesised causal relationships between applicant demographics, interview performance and offer outcomes in the ST3 selection process. This approach identified age, gender, ethnicity and country of primary medical qualification as the minimally sufficient adjustment set, with analyses across all six surgical specialties, and specialty included as a fixed effect to account for structural differences across recruitment pathways. A pooled DAG-adjusted model including all specialties with specialty included as a fixed effect was also generated for comparison (online supplemental table S3).

Data were analysed using R statistical software (V.2024.12.1+563).^19^ Descriptive statistics were presented using proportions and summary measures, as appropriate. Univariate analysis was performed to assess associations between each predictor variable and the primary outcome of offer made status. ORs with 95% CIs were calculated for each variable. The following reference categories were used: UK graduates (for country of qualification), younger applicants below the mean age (for age), White ethnicity (for ethnicity) and male (including trans male) for gender. Age was dichotomised at the mean within each specialty to improve interpretability and model stability in smaller subgroups; exploratory modelling using age as a continuous variable yielded consistent findings. Linearity of continuous variables was assessed, and diagnostic results for age are reported in online supplemental table S2. A p value of <0.05 was considered statistically significant for all analyses.

### Patient and public involvement

Patients and members of the public were not involved in the design, conduct, reporting or dissemination of this research. The study analysed anonymised administrative recruitment data; therefore, formal PPI input was not applicable. No funding was required or accessed for this work.

## Results

A total of 2342 applications were submitted during the 2024 recruitment cycle. After excluding 332 duplicate applications from individuals applying to multiple surgical specialties, data from 2009 unique applicants were analysed. Overall, one applicant (0.05%) was excluded from modelling due to a missing value for country of primary qualification in the plastic surgery dataset. All other specialties had complete data.

**Figure 1:**
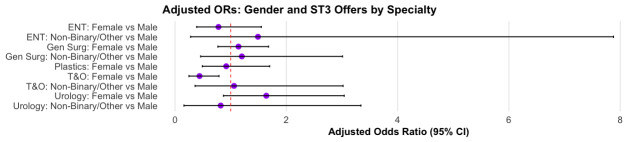
Forest plot illustrating gender-specific OR for receiving an ST3 surgical training job offer, stratified by speciality. Adjusted ORs (adjusted for age, ethnicity and country of primary medical qualification) are presented for ENT, plastic surgery, urology, general surgery and T&O. The non-binary/other versus male comparison is not shown for plastic surgery due to small numbers (<5) and unstable estimates. T&O, trauma and orthopaedics.

**Figure 2:**
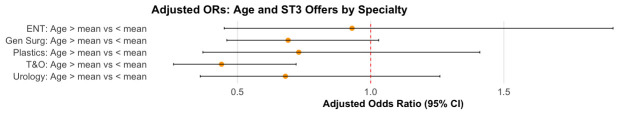
Forest plot showing OR for receiving an ST3 training job offer in applicants older than the mean age, stratified by specialty. Adjusted ORs (adjusted for gender, ethnicity and country of primary medical qualification) are presented for ENT, plastic surgery, urology, general surgery and T&O. T&O, trauma and orthopaedics.

**Figure 3:**
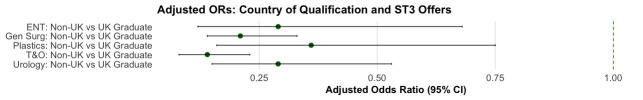
Forest plot illustrating OR of receiving an ST3 training job offer for non-UK/RoI graduates compared with UK/RoI graduates, stratified by specialty. Adjusted ORs (adjusted for age, ethnicity and gender) are presented for ENT, plastic surgery, urology, general surgery and T&O. T&O, trauma and orthopaedics.

Applicant characteristics are summarised in table 1. Across the six surgical specialties (ENT, plastic surgery, urology, paediatric surgery, T&O and general surgery) – female representation ranged from 15.7% in T&O to 47.7% in plastic surgery. The majority of applicants were UK or Ireland (UK/RoI) graduates (overall ~51%), though this varied by specialty. Median applicant age was 33.0 years (IQR 31.3 to 36.1) with minimal variation across specialties. White ethnicity was most common overall, but with substantial diversity in applicant pools, particularly in general surgery and T&O. Odds ratios (OR) by gender (figure 1), age (figure 2) and country of qualification (CoQ) (figure 3) demonstrate that demographic factors differentially impacted offer rates across specialties (table 2).

**Table 1:** Demographic summary of applicants and offers made for ST3 surgical training posts across six surgical specialities in 2024, highlighting distributions by gender, age, UK or Republic of Ireland medical school graduates and ethnicity

	ENT	Plastic surgery	Urology	General surgery	T&O	Paediatric surgery
Total applicants (n)	173	249	299	682	522	84
Offers made (n, %)	62 (35.8%)	61 (24.5%)	74 (24.7%)	204 (30.0%)	172 (33.0%)	9 (10.7%)
Gender (% applicants), n (% offers)						
Male	99 (57.2%) 36 (58.1%)	128 (51.4%) 30 (49.2%)	211 (70.6%) 43 (58.1%)	425 (62.4%) 107 (52.5%)	417 (79.9%) 135 (78.5%)	41 (48.8%) 1 (11.1%)
Female	66 (38.2%) 22 (35.5%)	118 (47.7%) 28 (45.9%)	75 (25.1%) 28 (37.8%)	228 (33.5%) 86 (42.2%)	82 (15.7%) 28 (16.3%)	39 (46.4%) 8 (88.9%)
Non-binary/other	8 (4.6%) 4 (6.5%)	3 (1.2%) 3 (4.9%)	13 (4.3%) 3 (4.1%)	28 (4.1%) 11 (5.4%)	23 (4.4%) 9 (5.2%)	4 (4.8%) 0 (0.0%)
Age						
Median age (IQR applicants; offers)	32 (30.4 to 34.1) 31.4 (30.3 to 33.5)	33 (31.1 to 35.5) 31.7 (30.5 to 34.1)	33.3 (31.4 to 35.9) 32.3 (31.2 to 34.3)	33.5 (31.1 to 36.5) 32.4 (30.5 to 34.4)	32.5 (30.6 to 35.3) 30.8 (30.1 to 32.6)	33.8 (31.2 to 37.3) 32.6 (31.2 to 33.6)
UK/RoI graduates (% applicants), n (% offers)						
UK graduates	125 (72.3%) 53 (85.5%)	156 (62.6%) 50 (82.0%)	124 (41.5%) 50 (67.6%)	198 (29.1%) 112 (54.9%)	219 (42.0%) 131 (76.2%)	29 (34.5%) 6 (66.7%)
Ethnicity n (% applicants), n (% offers)						
White	58 (33.5%) 20 (32.3%)	115 (46.2%) 34 (55.7%)	93 (31.3%) 29 (39.2%)	163 (23.9%) 65 (31.9%)	172 (33.0%) 80 (46.5%)	25 (29.8%) 4 (44.4%)
Asian	54 (31.2%) 20 (32.3%	51 (20.5%) 11 (18%)	85 (28.4%) 22 (29.7%)	238 (34.9%) 74 (36.3%)	158 (30.3%) 52 (30.2%)	22 (26.2%) 3 (33.3%)
Black	17 (9.8%) 5 (8.1%)	19 (7.6%) 3 (4.9%)	41 (13.7%) 6 (8.1%)	81 (11.9%) 15 (7.4%)	61 (11.7%) 10 (5.8%)	16 (19.0%) 0 (0.0%)
Mixed/multiple	3 (1.7%) 1 (1.6%)	5 (2%) 0 (0%)	8 (2.7%) 1 (1.4%)	25 (3.7%) 5 (2.5%)	10 (1.9%) 4 (2.3%)	3 (3.6%) 1 (11.1%)
OtherChinese	41 (23.7%) 16 (25.8%)	59 (23.7%) 13 (21.3%)	72 (24.1%) 16 (21.6%)	174 (25.6%) 45 (22.1%)	121 (23.3%) 26 (15.1%)	18 (21.4%) 1 (11.1%)

Percentages represent the proportion within each speciality’s applicant and successful offer pools, respectively. Age is reported as median with IQRs.

T&O, trauma and orthopaedics.

**Table 2:** Unadjusted and adjusted ORs with 95% CIs for receiving ST3 surgical training posts across various specialties, stratified by gender, age, ethnicity and country of primary medical qualification

	NTN offer: no offer	% ST3 job offer	Unadjusted OR (95% CI)	P value	Adjusted OR (95% CI)	P value
ENT (n=173)						
Gender						
Male	36:63	36.4	1	–	–	–
Female	22:44	33.3	0.88 (0.45 to 1.68)	0.700	0.78 (0.39 to 1.55)	0.477
Non-binary/other	4:4	50.0	1.75 (0.39 to 7.81)	0.448	1.49 (0.28 to 7.88)	0.631
Age						
<mean	42:66	38.9	1	–	–	–
>mean	20:45	30.8	0.70 (0.36 to 1.33)	0.282	0.93 (0.45 to 1.91)	0.847
Ethnic origin						
White	20:38	34.5	1	–	–	–
Asian	20:34	37.0	1.12 (0.51 to 2.43)	0.778	1.29 (0.56 to 2.96)	0.551
Black	5:12	29.4	0.79 (0.23 to 2.47)	0.697	1.24 (0.33 to 4.28)	0.742
Mixed/multiple	1:2	33.3	0.95 (0.04 to 10.51)	0.967	1.75 (0.07 to 23.03)	0.677
Other/Chinese	16:25	39.0	1.22 (0.53 to 2.79)	0.644	1.33 (0.52 to 3.43)	0.549
Country of qualification						
UK graduate	53:72	42.4	1	–	–	–
Non-UK grad	9:39	18.8	0.31 (0.13 to 0.68)	0.005	0.29 (0.12 to 0.68)	0.006
Plastic surgery (n=249)						
Gender						
Male	30:98	23.4	1	–	1	–
Female	28:90	23.7	1.02 (0.56 to 1.83)	0.957	0.92 (0.49 to 1.70)	0.779
Non-binary/other	3:0	100.0	~0 (NA to NA)	0.984	~0 (NA to NA)	0.990
Age						
<mean			1	–	1	–
>mean			0.51 (0.27 to 0.94)	0.033	0.73 (0.37 to 1.41)	0.349
Ethnic origin						
White	34:81	29.6	1	–	1	–
Asian	11:40	21.6	0.66 (0.29 to 1.39)	0.287	0.62 (0.26 to 1.38)	0.260
Black	3:16	15.8	0.45 (0.10 to 1.45)	0.223	0.66 (0.14 to 2.29)	0.545
Mixed/multiple	0:5	0	~0 (NA to NA)	0.988	~0 (NA to NA)	0.988
Other/Chinese	13:46	22.0	0.67 (0.31 to 1.38)	0.291	0.73 (0.32 to 1.59)	0.433
Country of qualification						
UK graduate	50:106	32.1	1	–	1	–
Non-UK grad	11:81	12.0	0.29 (0.13 to 0.57)	<0.001	0.36 (0.16 to 0.75)	0.009
Urology (n=299)						
Gender						
Male	43:168	20.4	1	–	1	–
Female	28:47	37.3	2.33 (1.30 to 4.14)	0.004	1.64 (0.87 to 3.04)	0.121
Non-binary/other	3:10	23.1	1.17 (0.25 to 4.03)	0.815	0.82 (0.16 to 3.34)	0.792
Age						
<mean	54:122	30.7	1	–	1	–
>mean	20:103	16.3	0.44 (0.24 to 0.77)	0.005	0.68 (0.36 to 1.26)	0.222
Ethnic origin						
White	29:64	31.2	1	–	1	–
Asian	22:63	25.9	0.77 (0.40 to 1.48)	0.435	1.22 (0.59 to 2.51)	0.590
Black	6:35	14.6	0.38 (0.13 to 0.95)	0.050	0.74 (0.24 to 2.04)	0.581
Mixed/multiple	1:7	12.5	0.32 (0.02 to 1.89)	0.291	0.41 (0.02 to 2.83)	0.440
Other/Chinese	16:56	22.2	0.63 (0.31 to 1.27)	0.202	0.97 (0.43 to 2.16)	0.939
Country of qualification						
UK graduate	50:74	40.3	1	–	1	–
Non-UK grad	24:151	13.7	0.24 (0.13 to 0.41)	<0.001	0.29 (0.15 to 0.53)	<0.001
General surgery (n=682)						
Gender						
Male	107:318	25.2	1	–	1	–
Female	86:142	37.7	1.80 (1.27 to 2.55)	<0.001	1.14 (0.77 to 1.68)	0.500
Non-binary/other	11:17	39.3	1.92 (0.85 to 4.19)	0.104	1.20 (0.46 to 3.01)	0.702
Age						
<mean	148:239	38.2	1	–	1	–
>mean	59:238	19.0	0.38 (0.26 to 0.54)	<0.001	0.69 (0.46 to 1.03)	0.067
Ethnic origin						
White	65:98	39.9	1	–	1	–
Asian	74:164	31.1	0.68 (0.45 to 1.03)	0.070	1.24 (0.77 to 2.00)	0.384
Black	15:66	18.5	0.34 (0.18 to 0.64)	0.175	0.77 (0.37 to 1.53)	0.463
Mixed/multiple	5:20	20.0	0.38 (0.12 to 0.99)	0.121	0.82 (0.25 to 2.31)	0.717
Other/Chinese	45:129	25.9	0.53 (0.33 to 0.83)	0.330	0.82 (0.47 to 1.41)	0.474
Country of qualification						
UK graduate	112:86	56.5	1	–	1	–
Non-UK grad	92:391	19.0	0.18 (0.13 to 0.26)	<0.001	0.21 (0.14 to 0.33)	<0.001
T+O (n=522)						
Gender						
Male	135:282	32.4	1	–	1	–
Female	28:54	34.1	1.08 (0.65 to 1.77)	0.754	0.44 (0.25 to 0.79)	0.006
Non-binary/other	9:14	39.1	1.34 (0.55 to 3.14)	0.503	1.06 (0.36 to 3.02)	0.913
Age						
<mean	141:171	45.2	1	–	1	–
>mean	31:179	14.8	0.21 (0.13 to 0.32)	<0.001	0.44 (0.26 to 0.72)	0.001
Ethnic origin						
White	80:92	46.5	1	–	1	–
Asian	52:106	32.9	0.56 (0.36 to 0.88)	0.012	0.89 (0.52 to 1.53)	0.679
Black	10:51	16.4	0.23 (0.10 to 0.46)	<0.001	0.73 (0.30 to 1.67)	0.468
Mixed/multiple	4:6	40.0	0.77 (0.19 to 2.78)	0.689	1.77 (0.36 to 7.87)	0.464
Other/Chinese	26:95	21.5	0.13 (0.18 to 0.53)	<0.001	0.53 (0.27 to 1.02)	0.060
Country of qualification						
UK graduate	131:88	59.8	1	–	1	–
Non-UK grad	41:262	13.5	0.11 (0.07 to 0.16)	<0.001	0.14 (0.08 to 0.23)	<0.001

Age dichotomised at the within-specialty mean (>mean vs <mean). Multivariate analysis was performed for ENT, plastic surgery, urology, general surgery and trauma and orthopaedics, adjusting for significant variables identified in univariate analyses. Statistically significant findings (p<0.05) are highlighted, notably demonstrating consistently lower odds of offer success for non-UK graduates across all specialties.

OR by gender (figure 1) showed that women had an equal chance of an offer as men, except in T&O (adjusted OR (aOR)=0.44, 95% CI 0.25 to 0.79, p=0.006) (table 2). Age (figure 2) was significantly associated with offers made in T&O, where applicants older than the mean age had significantly lower odds of receiving an offer compared with younger applicants (aOR=0.44, 95% CI 0.26 to 0.72, p=0.001; table 2). Across all specialties analysed, UK or Ireland (UK/RoI) medical graduates consistently demonstrated significantly higher odds of receiving an ST3 job offer compared with international medical graduates (figure 3; table 2). A pooled DAG-adjusted model showed the same pattern, with non-UK qualification remaining the dominant factor associated with lower offer odds (online supplemental table S3).

### Sensitivity analysis

A secondary analysis was conducted using ‘offer accepted’ as the outcome to evaluate robustness of the primary findings. Results were consistent with the primary analysis, with no change in the direction or significance of associations for key demographic predictors, including CoQ, age and gender. Full comparative results are available in online supplemental table S1.

### ENT (otolaryngology)

Among 173 applicants, 62 received job offers (35.8%) (table 1). Applicants were predominantly male (including trans male, 57.2%), 38.2% were female (including trans female), with a small proportion identifying as non-binary or other. UK/RoI medical graduates made up 72.3% of the ENT applicants. The most common ethnic origins were White British (n=39), followed by Asian or Asian British—Indian (n=26) with a wide distribution observed across multiple other ethnic groups. In the multivariable model adjusting for age, gender and ethnicity - non-UK/RoI graduates had markedly lower odds of receiving an offer compared with UK/RoI graduates (aOR 0.29, 95% CI 0.12 to 0.68, p=0.006). Gender and age were not associated with offer success, and ethnicity showed no independent effect after adjustment (table 2).

### Plastic surgery

Among 249 applicants, 61 were offered ST3 jobs (24.5%) (table 1). The cohort had a balanced gender representation (51.4% male, 46.8% female). Notably, non-UK/RoI graduates had significantly lower odds (63% reduction) of being offered an ST3 job compared with UK/RoI graduates (aOR=0.36, 95% CI 0.16 to 0.75, p=0.009) (table 2). Gender, ethnicity, and age were not significantly associated with outcomes, nor was age, although it showed a non-significant trend toward lower odds in older applicants (aOR=0.73, 95% CI 0.37 to 1.41, p=0.349).

### Urology

There were 299 applicants to urology, of whom 74 received offers (24.7%) (table 1). Most applicants identified as male (70.6%), and over half (58.5%) were international medical graduates. CoQ significantly associated with job offer likelihood; international medical graduates had approximately 71% lower odds compared with UK/RoI graduates (aOR=0.29, 95% CI 0.15 to 0.53, p<0.001) (table 2). Despite female applicants showing higher odds in unadjusted analysis, gender did not remain significant after adjustment (aOR=1.64, 95% CI 0.87 to 3.04, p=0.121).

### General surgery

General surgery received the highest number of applicants (682) with 204 applicants receiving job offers (29.9%) (table 1). International medical graduates had 79% lower odds of receiving offers compared with UK/RoI graduates (aOR=0.21, 95% CI 0.14 to 0.33, p<0.001) (table 2). Gender, age and ethnicity were not significantly associated with outcome in the multivariate analyses.

### Trauma and orthopaedics

Of 522 applicants for T&O, 172 were offered ST3 posts (33.0%) (table 1). Applicants identifying as female had a highly significant reduction in the odds of receiving an offer of a national training number compared with male counterparts (aOR=0.44, 95% CI 0.25 to 0.79, p=0.006) (table 2). Older applicants (>mean age) also had significantly lower odds (56% reduction) compared with younger peers (aOR=0.44, 95% CI 0.26 to 0.72, p=0.001). International medical graduates (IMGs) had lower odds (86%) of being offered an NTN compared with UK/RoI graduates (aOR=0.14, 95% CI 0.08 to 0.23, p<0.001), the largest observed reduction in T&O based on CoQ (table 2).

### Paediatric surgery

A total of 84 candidates applied to Paediatric Surgery, of whom 9 received offers (10.7%) (table 1). Almost half of applicants were female (including trans female) (48.1%), and 6 of the nine successful applicants were UK graduates (table 1). The median age of successful applicants was 32 years (IQR 30.0 to 34.5). Ethnically, the applicant pool was diverse, with the largest groups being ‘Black or Black British – African’ (n=16), ‘Other ethnic groups – any other ethnic group’ (n=10), and ‘White – any other White background’ (n=9). Despite this diversity among applicants, successful candidates were less varied. Due to the small sample size, statistical analysis to identify significant associations with demographic factors was not feasible.

## Discussion

This study provides a comprehensive analysis of equity in the 2024 ST3 national selection process across surgical specialties. UK graduates had a structural advantage across all specialties, likely reflecting familiarity with NHS practice, assessments and interview styles. This overall pattern was also evident in the pooled DAG-adjusted model (online supplemental table S3) although pooling across specialties inevitably averages out genuine variation and may obscure where targeted support is most needed. These associations should be interpreted as indicative rather than causal, as residual confounding cannot be fully excluded despite DAG-informed adjustment.

Age emerged as an independent predictor in trauma and orthopaedics, with older applicants having significantly lower odds of receiving an offer. Older trainees may have longer or non-linear career trajectories; however, the dataset did not include details of applicants’ previous posts (ie, training post, locally employed doctor or SAS). Gender differences were only statistically significant in trauma and orthopaedics, where female applicants had substantially lower odds of receiving a National Training Number compared with their male counterparts. This aligns with prior UK evidence showing persistent under-representation and differential attainment for women in orthopaedics, suggesting that targeted action may be needed to address structural and cultural barriers.^9 10 20^

It is well documented that IMGs face barriers in training, including challenges accessing training posts, passing postgraduate examinations and progressing through training pathways.^21^,^23^ IMGs frequently have limited access to mentorship, unfamiliarity with NHS systems and expectations, cultural and communication difficulties, and a lack of tailored preparation for UK-specific assessments such as the Membership of the Royal Colleges of Surgeons (MRCS) examination and portfolio-based interviews.^21^,^23^ Studies consistently demonstrate that IMGs require prolonged adjustment periods to adapt to clinical, cultural and organisational expectations within the NHS, potentially resulting in reduced confidence, weaker performance in scenario-based interviews or unfamiliarity with portfolio expectations.^2123^,^25^ Consequently, the clear and consistent advantage observed among UK-trained applicants is likely linked to familiarity with NHS practices, clinical communication expectations and interview formats.^25 26^ Targeted interventions, such as NHS-specific induction programmes, focused interview preparation courses and structured targeted mentorship, designed to support equitable access to training posts, might mitigate some of these challenges.^21 23 25^ However, lower odds of appointment are unrelated to ethnicity, which was not a significant factor in chances of an offer being made.

Other studies report disparities in ARCP outcomes, MRCS exam performance and career satisfaction. The lack of significant associations for ethnicity or gender in most specialties suggests current selection processes may reduce overt bias but cannot address structural barriers.^9 13 20^ Additionally, small applicant numbers in some specialties may limit statistical power to detect disparities. Additionally, the use of broad ethnic categories, such as Asian, while aligned with ONS guidance, may obscure important within-group variation. Outcomes could differ between Indian, Pakistani or Chinese applicants, so disaggregated analysis should be prioritised in future studies to better understand these patterns.

While not directly assessed in this study, initiatives by surgical Royal Colleges to promote inclusivity and mentorship may play a role in shaping more equitable recruitment environments. Such initiatives help build confidence, provide guidance on navigating the application process and increase access to support networks for minoritised groups.^9 24 26^ Although anonymised, standardised selection processes appear to support more equitable shortlisting, anonymisation alone cannot fully address deeper structural inequalities such as differential access to experience, professional networks or prior opportunities.^22 27^

This study’s strength is that it analyses data from a large, multispecialty national dataset. It is important that timely reports are available so that appropriate interventions can be made if differential attainment is found to minimise the risk in the following year. The dataset lacked data on socioeconomic indicators and disability. Socioeconomic background is not a protected characteristic but does impact on performance in postgraduate exams.^28^

Qualitative research with surgically inclined IMGs and applicants from minoritised backgrounds could provide valuable insight into perceived and experienced barriers in the selection process. Continued monitoring of equity outcomes in surgical recruitment, particularly in relation to ethnicity and intersectional identity, is required. Although our analysis focused on individual characteristics due to power constraints, we acknowledge that intersecting identities, such as being an older, female IMG, may confer compounded disadvantage. Future studies should explore these intersectional effects using larger datasets or qualitative methods to better understand cumulative barriers.

Finally, specialty-specific interventions, such as tailored induction, mentorship and preparatory resources, may be needed to support non-UK graduates in navigating the selection process and enhancing equitable access to higher surgical training. Rasch analysis minimises differential attainment by ensuring that all candidates are assessed on the same scale and adjusting for ‘differential item functioning’, correcting for unconscious biases.^29^ It has been used previously in General Surgery selection, but could helpfully be reintroduced across all processes, and may be somewhere where artificial intelligence (used as appropriately trained human statisticians are scarce) might be employed to augment equity.

## Conclusion

UK graduates have a clear advantage in ST3 surgical selection. Efforts to improve equity should focus on support for IMGs and ongoing review of structural barriers. Current anonymised processes appear to mitigate some bias but are insufficient alone.

## Supplementary material

10.1136/bmjopen-2025-106487 online supplemental file 1

## Data Availability

Data are available upon reasonable request.
