# Supplementary material for: Fair access to higher surgical training in the UK: an equity, diversity and inclusion analysis of national selection in 2024
Source: BMJ Open. 2025 Nov 13;15(11):e106487. doi: 10.1136/bmjopen-2025-106487 (PMC12625904; doi:10.1136/bmjopen-2025-106487)
Supplement: online supplemental file 1 [file bmjopen-15-11-s001.docx]

Supplementary Table S1: Primary analyses modelled the odds of receiving an offer (“Offer Made”); sensitivity analyses modelled the odds of accepting an offer (“Offer Accepted”). Models were stratified by specialty and run separately for each subgroup, with demographic predictors entered individually. Variables include gender (male = reference), age (≤ mean = reference), ethnicity (White = reference), and country of primary medical qualification (UK graduate = reference). Adjusted models included all demographic variables simultaneously. Missing or sparse categories (–) reflect model non-convergence due to small cell counts.

|  | Primary Analysis  Offer Made  (unadjusted OR, 95% CI) | Sensitivity Analysis  Offer Accepted (unadjusted OR, 95% CI) |
| --- | --- | --- |
| **ENT (n = 173)** | n = 62 | n = 59 |
| Gender | | |
| Male | 1 | 1 |
| Female | 0.875 (0.45 – 1.68) | 1.00 (0.51 – 1.93) |
| Non-Binary / Other | 1.75 (0.39 – 7.81) | 2.00 (0.45 – 8.95) |
| Age | | |
| <mean | 1 | 1 |
| >mean | 0.70 (0.36 – 1.33) | 0.63 (0.32 – 1.21) |
| Ethnic Origin | | |
| White | 1 | 1 |
| Asian | 1.12 (0.51 – 2.43) | 1.21 (0.55 – 2.67) |
| Black | 0.79 (0.23 – 2.47) | 0.93 (0.26 – 2.91) |
| Mixed / Multiple | 0.95 (0.04 – 10.51) | 1.11 (0.05 – 12.34) |
| Other / Chinese | 1.22 (0.53 – 2.79) | 1.42 (0.61 – 3.30) |
| Country of Qualification | | |
| UK graduate | 1 | 1 |
| Non-UK graduate | 0.31 (0.13 – 0.68) | 0.35 (0.15 – 0.75) |
|  | | |
|  | **Primary Analysis**  **Offer Made**  **(adjusted OR, 95% CI)** | **Sensitivity Analysis**  **Offer Accepted (adjusted OR, 95% CI)** |
| **Plastic Surgery (n = 249)** | n = 61 | n = 60 |
| Gender | | |
| Male | 1 | 1 |
| Female | 0.90 (0.48 – 1.66) | 0.93 (0.50 – 1.73) |
| Non-Binary / Other | - (0 – NA) | - (0 – NA) |
| Age | | |
| <mean | 1 | 1 |
| >mean | 0.72 (0.37 – 1.39) | 0.67 (0.34 – 1.30) |
| Ethnic Origin | | |
| White | 1 | 1 |
| Asian | 0.62 (0.26 – 1.38) | 0.65 (0.27 – 1.43) |
| Black | 0.66 (0.14 – 2.28) | 0.69 (0.15 – 2.39) |
| Mixed / Multiple | - (NA) | - (NA) |
| Other / Chinese | 0.73 (0.32 – 1.59) | 0.76 (0.33 – 1.67) |
| Country of Qualification | | |
| UK graduate | 1 | 1 |
| Non-UK graduate | 0.37 (0.16 – 0.77) | 0.39 (0.17 – 0.81) |
|  | | |
| **Urology (n = 299)** | n = 74 | n = 70 |
| Gender | | |
| Male | 1 | 1 |
| Female | 1.62 (0.86 – 2.99) | 1.36 (0.71 – 2.56) |
| Non-Binary / Other | 0.89 (0.17 – 3.57) | 0.83 (0.16 – 3.39) |
| Age | | |
| <mean | 1 | 1 |
| >mean | 0.67 (0.36 – 1.25) | 0.63 (0.33 – 1.18) |
| Ethnic Origin | | |
| White | 1 | 1 |
| Asian | 1.21 (0.59 – 2.50) | 1.26 (0.61 – 2.62) |
| Black | 0.74 (0.24 – 2.04) | 0.68 (0.20 – 1.95) |
| Mixed / Multiple | 0.41 (0.02 – 2.83) | 0.47 (0.02 – 3.22) |
| Other / Chinese | 0.97 (0.43 – 2.16) | 1.07 (0.47 – 2.41) |
| Country of Qualification | | |
| UK graduate | 1 | 1 |
| Non-UK graduate | 0.29 (0.15 – 0.53) | 0.27 (0.14 – 0.51) |
|  | | |
| **General Surgery (n = 682)** | n = 204 | n = 184 |
| Gender | | |
| Male | 1 | 1 |
| Female | 1.14 (0.77 – 1.68) | 1.09 (0.74 – 1.61) |
| Non-Binary / Other | 1.20 (0.47 – 2.99) | 1.01 (0.38 – 2.55) |
| Age | | |
| <mean | 1 | 1 |
| >mean | 0.69 (0.46 – 1.03) | 0.72 (0.48 – 1.08) |
| Ethnic Origin | | |
| White | 1 | 1 |
| Asian | 1.24 (0.77 – 2.00) | 1.24 (0.77 – 2.01) |
| Black | 0.77 (0.37 – 1.53) | 0.88 (0.43 – 1.74) |
| Mixed / Multiple | 0.82 (0.25 – 2.31) | 0.70 (0.19 – 2.07) |
| Other / Chinese | 0.82 (0.47 – 1.41) | 0.88 (0.50 – 1.52) |
| Country of Qualification | | |
| UK graduate | 1 | 1 |
| Non-UK graduate | 0.21 (0.14 – 0.33) | 0.26 (0.17 – 0.40) |
|  | | |
| **Trauma and Orthopaedics**  **(n = 522)** | n = 172 | n = 164 |
| Gender | | |
| Male | 1 | 1 |
| Female | 0.45 (0.24 – 0.79) | 0.45 (0.25 – 0.79) |
| Non-Binary / Other | 1.04 (0.36 – 2.91) | 0.94 (0.32 – 2.61) |
| Age | | |
| <mean | 1 | 1 |
| >mean | 0.44 (0.26 – 0.73) | 0.44 (0.26 – 0.73) |
| Ethnic Origin | | |
| White | 1 | 1 |
| Asian | 0.89 (0.52 – 1.53) | 0.83 (0.49 – 1.42) |
| Black | 0.73 (0.30 – 1.67) | 0.71 (0.29 – 1.62) |
| Mixed / Multiple | 1.77 (0.36 – 7.87) | 1.79 (0.37 – 7.80) |
| Other / Chinese | 0.53 (0.27 – 1.02) | 0.50 (0.26 – 0.95) |
| Country of Qualification | | |
| UK graduate | 1 | 1 |
| Non-UK graduate | 0.14 (0.08 – 0.23) | 0.16 (0.10 – 0.27) |

Supplementary Figure S1: Directed Acyclic graph (DAG) for confounder selection. The DAG depicts hypothesised relationships influencing the probability of receiving an ST3 offer. Demographic variables (gender, age, ethnicity, country of primary qualification) may influence interview or portfolio performance and the selection decision. Specialty affects both assessment structure and selection thresholds. Unmeasured factors such as socioeconomic background, UK clinical experience and English proficiency may confound these relationships. Interview or portfolio performance lies on the causal pathway and therefore not adjusted for. The minimally sufficient adjustment set for each demographic exposure includes the other demographic variables, with specialty entered as a fixed effect.


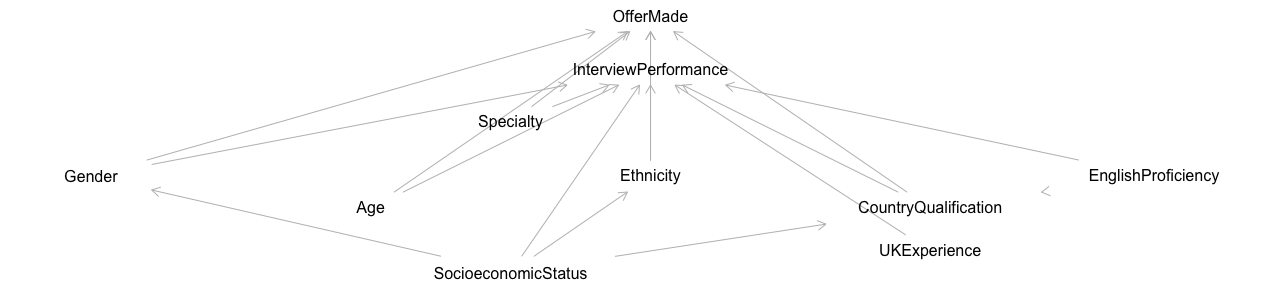


Supplementary Table S2: assessment of linearity of age on the logit scale (pooled dataset)

| Test | Model specification | Test statistic | df | p-value | Interpretation |
| --- | --- | --- | --- | --- | --- |
| Box-Tidwell test | Age x log(Age) term in the pooled model (adjusted for gender, ethnicity, CoQ, specialty) | Estimate = -0.64 (SE = 0.32),  z = -1.97 | 1 | 0.049 | Mild deviation from linearity |
| Likelihood-ratio test | Linear vs. spline (df = 3) age terms | ΔDeviance = 5.82 | 2 | 0.055 | Borderline improvement in fit for spline |
| Visual check (Figure S1) | LOESS smoother of empirical logits (OfferMade ~ Age) | - | - | - | Gentle curvature, slight U shape at mid-to-later ages |

Supplementary Figure S2: Pooled LOESS smoother of empirical log-odds of receiving an offer by age (weights ∝ bin size) The curve shows mild curvature, consistent with Box-Tidwell (p = 0.049) and LRT linear vs spline (p = 0.055) results, suggesting only borderline non-linearity


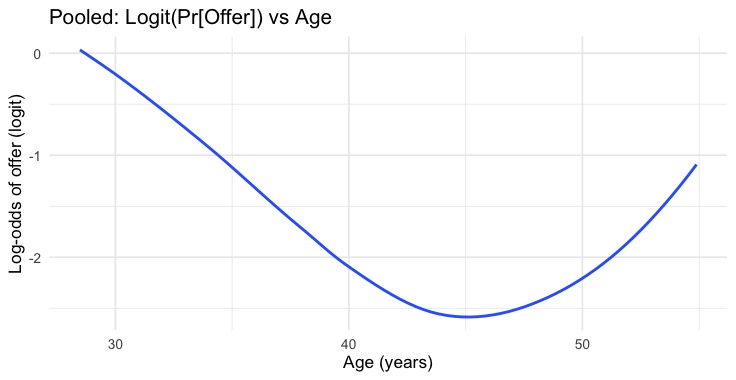


Supplementary Table S3: Pooled multivariable logistic regression including all five modelled specialties (ENT, plastic surgery, urology, general surgery, and T&O). The model adjusted for age (>mean vs <mean), gender, ethnicity, and country of primary qualification, with specialty entered as a fixed effect.

|  | Pooled analysis  Adjusted OR (95% CI) | P value |
| --- | --- | --- |
| Gender | | |
| Male | 1 | - |
| Female | 0.92 (0.72 – 1.17) | 0.514 |
| Non-binary/Other | 1.29 (0.74 – 2.24) | 0.360 |
| Age | | |
| <mean | 1 | - |
| >mean | 0.62 (0.49 – 0.79) | <0.001 |
| Ethnicity | | |
| White | 1 | - |
| Asian | 1.05 (0.80 – 1.37) | 0.745 |
| Black | 0.75 (0.49 – 1.13) | 0.180 |
| Mixed / Multiple | 0.77 (0.35 – 1.57) | 0.496 |
| Other / Chinese | 0.76 (0.55 – 1.04) | 0.083 |
| Country of Qualification | | |
| UK graduate | 1 | - |
| Non-UK graduate | 0.21 (0.16 – 0.27) | <0.001 |
