# Supplementary material for: Fair access to higher surgical training in the UK: an equity, diversity and inclusion analysis of national selection in 2024
Source: BMJ Open. 2025 Nov 13;15(11):e106487. doi: 10.1136/bmjopen-2025-106487 (PMC12625904; doi:10.1136/bmjopen-2025-106487)
Supplement: Supplementary file 2 [file bmjopen-2025-106487.reviewer_comments.pdf]

PEER REVIEW HISTORY

BMJ Open publishes all reviews undertaken for accepted manuscripts. Reviewers are asked to complete a checklist review form and are provided with free text boxes to elaborate on their assessment. These free text comments are reproduced below.

ARTICLE DETAILS

Title (Provisional)

Fair access to higher surgical training in the UK: an equity, diversity and inclusion analysis of national selection in 2024

Authors

Seehra, Jaspreet Kaur; Ellis, Ricky; Doleman, Brett; McLarty, Esther; Lund, Jonathan

---

VERSION 1 - REVIEW

**Reviewer**	**1**
**Name**	**Dack, Kyle**
**Affiliation**	**University of Bristol, MRC Integrative Epidemiology Unit**
**Date**	**22-Jul-2025**
**COI**	**None**

Statistical review summary:

I have two key concerns related to the statistical methodology. First, the model building strategy is based on statistical significance, which increases the risk of bias because this is primarily a method of building predictive models rather than models for inference. Second and related, this is a cross-sectional analysis but does not consider or discuss much the risk of confounding bias, which is especially important because the authors use causal language in the paper when describing the associations. I suggest using more cautious language and softer conclusions, or modifying the model building process to gear more towards minimizing confounding bias.

Abstract:

1. Participants: I suggest clarifying how many specialities were evaluated, because this impacts the interpretation of the results, e.g. the results subheading currently reports two specialties, which implies that in any other specialities the associations were not significant. But currently abstract readers will not know if there were any other specialities evaluated.

2. The final 95% CI has a typo: “CL” instead of “CI”.

Methods

3. Missing data: There is a comment at the end of the Study Population subheading which states that applicants without the variables of interest were excluded. I suggest reporting how many were excluded in the results, including if there were any particularly variables causing most of the exclusions.

4. Statistical analysis: It is very helpful that the authors report the reference categories, but it is hard to follow these without knowing the comparison categories. It looks as if age was converted to a categorical variable, which should be justified because this results in a loss of statistical information and possibly power to find an association.

5. My primary statistical concern relates to the construction of the multivariable model. Variable selection does not appear to account for confounding, overadjustment, or mediator bias, which is likely to limit the interpretability of the reported associations. It is unclear whether relevant confounders were identified, available in the dataset, or considered in the analysis. This is an important omission because confounding control is one of the primary items evaluated in risk of bias assessment tools, and the study is likely to be evaluated more positively if confounding bias is more explicitly addressed in the manuscript.

Additionally, the analysis uses a data-driven approach to variable selection, justified on the grounds of improving statistical power and model stability. However, excluding variables solely based on lack of univariate significance is unlikely to enhance power or stability, and the rationale for this claim is not fully stated. As the authors acknowledge, this approach may increase the risk of bias. Given that the paper is framed towards causal inference, a theory- or evidence-based selection of covariates would be more appropriate to ensure the associations can be interpreted confidently. The authors should either provide a stronger justification for the data-driven strategy or revise the model-building approach to align with inferential goals.

6. The manuscript reports subgroup analyses across different medical specialties. However, in the Outcomes, and Statistical Analysis sections, only a generic "offer made" status is described, without specifying the distinct specialties included. The completeness of the methodology could be improved by listing the medical specialties analysed in both sections, to ensure transparency and prevent readers from assuming a single outcome.

Results

7. Currently, the model results are reported before baseline characteristics. It would help readers to present baseline characteristics first, because these inform the interpretation of the model results. Currently table 1 is only referenced after sensitivity analysis – I suggest moving it to the first two paragraphs, and consider reporting the highlights in-text if there is space.

8. Figures 1-3. Suggest increasing the font size of the x-axis because it is easy to miss.

9. Figure 1. Why is there no comparison of non-binary/other vs male reported for plastics? If there is a limit due to low numbers I suggest adding this to the statistical analysis methods, and explaining in-text.

10. Figure 2. The authors could consider a sensitivity analysis of modelling age as a continuous measure (i.e. 1 year of age on odds of acceptance) to assess whether the categorization of age impacted the results. Or the use of median instead of mean, given that age may not be normally distributed. I also suggest reporting the mean age in the figure label to help readers understand the categories.

11. Table 2. The definition of the age categories should be reported to aid interpretability of these results.

12. Was an "overall" model including all specialities together considered? It would have significantly greater power, although I do not know how useful it is in terms of interpretation, but it may address the issue the authors note in the discussion regarding low power for ethnicity.

Discussion

13. My primary concern is the use of causal language in interpreting the results—for example, terms such as "predictor" and "influenced" in the abstract. While the study benefits from a clear temporal sequence, with characteristics measured prior to the outcome, potential confounding bias is not clearly addressed. As this is a key limitation of cross-sectional studies, integrating methods to minimise confounding bias, and discussing their effectiveness, are necessary for the causal language to be valid.

**Reviewer**	**2**
**Name**	**Nzenwa, Ikemsinachi**
**Affiliation**	**Hull York Medical School, Centre for Anatomical and Human Sciences**
**Date**	**28-Aug-2025**
**COI**	**None**

I read this manuscript with great interest. This is an important and timely study that addresses equity and diversity in surgical training selection within the UK. Seehra and colleagues should be commended for their rigorous analysis of a large national dataset and for focusing on an issue with significant implications for the surgical workforce. The findings are impactful, particularly the evidence that international medical graduates and female doctors face significant barriers in securing surgical training positions. These observations

will be of immediate interest to key stakeholders, including training program directors and advocacy groups.

I have a few minor comments and suggestions:

1. The sensitivity analysis was a thoughtful addition, as it highlights potential barriers that underrepresented doctors may face in accepting training positions.

2. It is reasonable to select variables for multivariable regression analyses based on clinical or socioeconomic relevance, rather than only those significant in univariate analyses. I suspect it would not change the results given the low sample size for study groups like ENT.

3. I recommend that, in the Discussion, you expand on the results of female doctors having significantly lower odds of ST3 selection. You should put this in context of other studies. It is an open secret but are your findings concordant with existing literature?

4. A careful proofreading of the manuscript is warranted to correct minor punctuation and grammar errors. For example, the penultimate line of the "Statistical Analysis" section, the misplaced period in the "Figure 2" legend, and the inconsistent use of the abbreviation "IMG." I would also suggest capitalizing the first letter in racial categories such as "White" and "Black".

VERSION 1 - AUTHOR RESPONSE

Reviewer 1 – Dr Kyle Dack

1) Comment: concerns regarding model-building strategy, confounding and causal language
   Response: we thank the reviewer for this valuable feedback. The *Statistical Analysis* section has been revised to describe a DAG-informed, confounder-driven model-building approach. A directed acyclic graph (Supplementary Figure S1) has been since developed identify key confounders and avoid over-adjustment and collider bias with a rerun of the modelling analysis
2) Comment 1&2: Abstract – clarify how many specialties & typo of 95% CL
   Response: we now state that six specialties were analysed in the Abstract and confirmed the 95% CI typography throughout

3) Comment 3: Methods – missing data: report exclusions
   Response: we now report missing data in the Results section, noting that only one record (0.055%) was excluded in plastic surgery due to missing country of primary qualification data. All other specialties had complete data
4) Comment 4: Statistical analysis – reference groups and dichotomy of age
   Response: we thank the reviewer for this observation. We have retained the categorical age variable for comparability across specialties and to maintain

model stability given smaller subgroup sizes. It also aids in interpretation of the coefficients for clinical readers, whom this paper is primarily targeted towards. A brief justification for this has been added to the *Statistical Analysis* section. Linearity checks using plots, Box-Tidwell and spline likelihood-ratio tests (reported in Supplementary Table S2 and Figure S1) showed deviation from linearity, confirming that inclusion of age as a continuous predictor was not appropriate.

5) Comment 5: model-building strategy and confounding control
   Response: we thank the reviewer for this important comment. The model-building approach has been revised to emphasise confounding control rather than statistical significance. Multivariate models were informed by a directed acyclic graph (DAG). This approach replaced the previous data-driven selection based on univariate significance. Age, gender, ethnicity and country of primary qualification were included as the minimally sufficient adjustment set, reflecting their theoretical evidence of their relevance to equity in surgical selection. The revised *Statistical Analysis* section and Supplementary Figure S1 now describe this confounder-driven framework explicitly.
   Following this change, all specialty analysis were re-run using the consistent DAG-assisted model. This update now includes a multivariable logistic regression for ENT, which was previously limited to univariate analysis. Revised *Results* and Table 2 now present adjusted odds ratios for ENT alongside other specialties.
6) Comment 6: The manuscript reports subgroup analyses across different medical specialties. However, in the Outcomes and Statistical Analysis sections, only a generic "offer made" status is described, without specifying the distinct specialties included.
   Response: we now explicitly mention the six surgical specialties analysed and reiterate the *Study population* in both *Outcomes* and *Statistical Analysis* subsections to ensure clarity and transparency.
7) Comment 7: Currently, the model results are reported before baseline characteristics. It would help readers to present baseline characteristics first, because these inform the interpretation of the model results.
   Response: Thank you for this useful suggestion. The *Results* section has been restructured so that baseline applicant characteristics (Table 1) are now presented in the opening paragraphs before the model results. We have also summarised key descriptive highlights in-text to guide interpretation.
8) Comment 8: Figures 1-3. Suggest increasing the font size of the x-axis because it is easy to miss.

Response: the x-axis and label font sizes in Figures 1 – 3 have been increased. All figures have been reformatted with larger test and clearer axis labels

9) Comment 9: figure 1 – missing comparison of non-binary/other vs male in plastic surgery
Response: we thank the reviewer for noticing this. The non-binary/other group in plastic surgery comprised only three applicants, none of whom received an offer, meaning the model could not estimate an odds ratio due to quasi-complete separation (as demonstrated in Table 2). This has been made clearer in the Figure 1 legend
10) Comment 10: modelling age as a continuous variable
Response: We agree and did consider this when running statistics on the original dataset. We conducted pooled linearity checks for age using a Box-Tidwell test ($p = 0.049$), a likelihood-ratio test comparing linear and spline age terms ($p = 0.055$) and a LOESS smoother of empirical logits (Figure S1). These analyses showed evidence of non-linearity. To maintain interpretability and model stability in smaller specialty-specific samples, we retained the dichotomised age variable in the main models. As our target audience are clinicians, we wanted to maximise interpretability, although we accept this comes at the cost of loss of information. Full results are provided in Supplementary Table S2 and Figure S2.
11) Comment 11: Table 2 – definition of age categories
Response: the definition of age categories has been clarified in the Table 2 legend
12) Comment 12: overall pooled analysis
Response: we have included a pooled DAG-adjusted logistic regression model incorporating all specialties (Supplementary Table S3). This pooled model confirms all overall associations observed in specialty-specific analyses, particular the strong impact of country of qualification. However, we retained specialty-level models as our primary focus because equity issues and selection structures differ across surgical specialties. Aggregating the data risks obscuring these genuine between-specialty variations, which are crucial for identifying where targeted support and policy interventions are most needed.
13) Comment 13: causal language in interpreting the results
Response: all causal language has been softened throughout the manuscript (eg replacing "predictor" with "factor associated with" and "influenced" with "associated with"). We have also clarified that this is an observational cross-sectional analysis and that no causal inference can be made.

Reviewer 2 - Mr. Ikemsinachi Nzenwa

1) Comment 1: The sensitivity analysis was a thoughtful addition, as it highlights potential barriers that underrepresented doctors may face in accepting training positions
Response: we thank the reviewer for this positive feedback. We have clarified in the *Study population* section that variables relating to visa or immigration status were unavailable in the dataset and explained how defining the outcome as “offer made” rather than “offer accepted” mitigates potential bias from right-to-work or visa restrictions.
2) Comment 2: It is reasonable to select variables for multivariable regression analyses based on clinical or socioeconomic relevance, rather than only those significant in univariate analyses. I suspect it would not change the results given the low sample size for study groups like ENT.
Response: agreed, thank you. We have adjusted the revised manuscript to adopt a confounder-driven, DAG-informed model (age, gender, ethnicity, CoQ) rather than univariate significance. We note this explicitly in *Statistical analysis* and retain specialty models despite smaller sample sizes (eg ENT) as results were unchanged
3) Comment 3: I recommend that, in the Discussion, you expand on the results of female doctors having significantly lower odds of ST3 selection. You should put this in context of other studies. It is an open secret but are your findings concordant with existing literature?
Response: we have added a short paragraph to the *Discussion* contextualising our T&O finding with existing literature, citing studies already in our reference list (e.g DE FACTO in T&O – reference 10) and broader disparities reviews (references 9 and 20)
4) Comment 4: proofreading / consistency (punctuation, legends, IMG, capitalisation of racial categories)
Response: we have carefully proofread and corrected minor errors and standardised terminology:
    - Defined IMG at first use and used consistently thereafter
    - Capitalised ethnic group categories (White, Black, Asian, Mixed/Multiple, Other/Chinese) throughout, aligning with ONS categorisation
    - Corrected punctuation/typos in legends and text

VERSION 2 - REVIEW

Reviewer 1

**Name**	**Dack, Kyle**
**Affiliation**	**University of Bristol, MRC Integrative Epidemiology Unit**
**Date**	**19-Oct-2025**
**COI**	

The revision has transparent methods, and uses reliable statistical methods to minimise bias. It looks like a really nice paper.

My only comment is that although the authors stated that causal language was removed from the manuscript, I still found a few instances of this such as "influenced" or "predictors". This may be something which could be resolved either in proofing or a minor revision.
